# A complex network analysis of ethnic conflicts and human rights violations

**DOI:** 10.1038/s41598-017-09101-8

**Published:** 2017-08-15

**Authors:** Kiran Sharma, Gunjan Sehgal, Bindu Gupta, Geetika Sharma, Arnab Chatterjee, Anirban Chakraborti, Gautam Shroff

**Affiliations:** 1 School of Computational and Integrative Sciences, Jawaharlal Nehru University, New Delhi, 110067 India; 2 TCS Research, New Delhi, India

## Abstract

News reports in media contain records of a wide range of socio-economic and political events in time. Using a publicly available, large digital database of news records, and aggregating them over time, we study the network of ethnic conflicts and human rights violations. Complex network analyses of the events and the involved actors provide important insights on the engaging actors, groups, establishments and sometimes nations, pointing at their long range effect over space and time. We find power law decays in distributions of actor mentions, co-actor mentions and degrees and dominance of influential actors and groups. Most influential actors or groups form a giant connected component which grows in time, and is expected to encompass all actors globally in the long run. We demonstrate how targeted removal of actors may help stop spreading unruly events. We study the cause-effect relation between types of events, and our quantitative analysis confirm that ethnic conflicts lead to human rights violations, while it does not support the converse.

## Introduction

The quantitative analyses of the gigantic amount of data associated with human social conditions have gained much momenta in the recent years, especially through the multidisciplinary tools and approaches. The traditional theories of social science together with complex network analysis^1–3^ and addition of new tools and paradigms from several science disciplines like theoretical physics, applied mathematics, computer science, as well as psychology, have developed into what is presently known as computational social science^4^ and have helped uncover new patterns of social behavior, including social dynamics^5, 6^. Data made available digitally, has drawn attention of researchers across disciplines who have collaborated and contributed in their own ways to scientifically analyze and understand complex social phenomena in the recent years, previously not known to the present scale of detail.

Social behavior, conditions, events, organizations, which have given shape to the history of human civilization, have often been positive or constructive – building societies, shaping cultures, and at other times negative or destructive – conflicts, wars and battles followed by reorganization of countries or nations. Human civilization has evolved in a complicated manner and created a variety in ethnic characters and cultures, in languages, beliefs and customs. It is widely understood^7^ that historically, certain groups pay priority to their linguistic, cultural, racial, and religious ties of individuals within the groups, which are then passed down through generations. Ethnic violence arises when groups attempt to maintain their boundaries against pressure from historical enemies. In another possible scenario, when the authority of a multi-ethnic state declines, the central regime ceases to protect the interests of ethnic groups, creating a void in which ethnic groups start competing to establish and control a new regime that will protect their interests. Socio-political tension in such a situation is likely to incite violence.

Ethnic heterogeneity is a characteristic feature observed in most countries and regions worldwide. With time, these variations often reduce locally, where socio-economic and political forces are less dominant in favor of ethnic mixing. However, the course of political history along with that of religion and culture often encounters conflicts at various scales. Ethnic conflicts are comparatively frequent in certain regions and rare elsewhere. Ethnic heterogeneity does not necessarily breed war and its absence does not ensure peace. Even today, ethnic wars continue to be globally the most common form of armed conflicts, but the mechanisms that lead a society down the path of ethnic conflict are yet to be fully understood. What is intriguing, is the connection between democratization and the occurrence of ethnic conflict. While stable democracies are unlikely to wage war with other democracies, a country that is socio-politically unstable may very well find conflicts between groups with opposing interests^8^. Human rights are internationally agreed values, standards or rules regulating the conduct of states towards their own citizens and towards non-citizens. Interestingly, the violation of human rights appears to be more associated with ethnic conflicts than abuses of economic and social rights^9^. Other political, economic and social preconditions may also influence the causes of ethnic conflicts, and the conscious promotion by the political actors of any polarizing dimension based on these factors is sufficient to lead to conflict. It is often seen that changes in political regime can lead to conflicts which are often ethnic in various parts of the world, sparking human rights violations. Therefore, spatio-temporal analyses of regional conflict formations and political dynamics, and the statistical studies of the different variables are important.

Temporal data of events like human to human communications and physical contacts/proximity has been studied in recent years (see e.g., Holme and Saramäki)^10^ in connection to study of epidemics and contagion, spreading of information using mobile phone communication data^11^ as well as data from proximity sensors carried by human entities^12^.

Here we provide a quantitative analysis of the scale and topology of ethnic tensions, related conflicts and violations of human rights, using the reports in digital media. We look at data from a publicly accessible database, which keeps account of events from news available in media. We particularly focus on ethnic conflicts (EC) and human rights violations (HR) by suitably filtering keywords present in the digital text transcript of the news. With the availability of high precision data containing precise spatio-temporal information, one can look towards finding correlations between events, involved actors (individuals, groups, organizations or states), the geographical pattern of spreading of conflicts etc. Although there have been studies on conflicts, wars and terror attacks^13–16^, and speculation of ethnic conflicts using census data for segregated population^17^, a comprehensive study of the actors involved in ethnic conflicts and human rights violation has been lacking. In this work, for the first time, we provide a quantitative and qualitative understanding of the relative activity of the actors, frequently engaging actor pairs, the network of actors, and give insights into the static and dynamical aspects of the actor network in a scenario involving ethnic conflicts and human rights violations.

## Results

GDELT Event Database^18^ contains the database of news articles from all over the world in several languages. Using a query, it is possible to extract data for events, about ethnic conflicts (EC) and human rights violations (HR) happening around the world, spanning over a large time scale. Each event data contains information such as the pair of *actors* involved, a unique time stamp, names of individuals, organizations or groups, the location information of the event, as well as latitude, longitude data of actors and the event. The news event and subsequently the data serves as a proxy for the actual event and its intensity (in terms of number of reports). Although we do not analyze the actual news reports, we consider a report on EC to be an actual instance of ethnic conflict or its possibility, and similarly for HR. We analyzed 28,055 events of EC and 36,470 of HR for a 15 year period (2001–2015).

**Figure 1:**
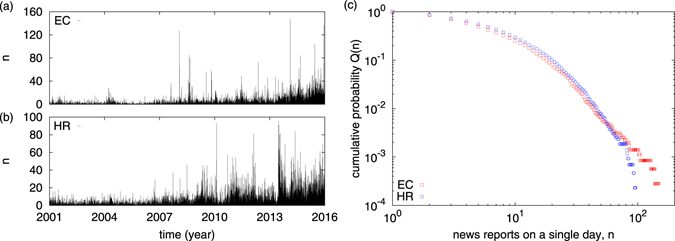
The time sequence of the number of events *n* reported daily for (**a**) EC, (**b**) HR during 2001–2015. (**c**) The cumulative probability (CCDF) *Q*(*n*) that *n* or more events are reported on a particular day. The data for EC seems to fit well to a power law for the largest values, with decay exponent around 2.54 ± 0.03, HR fits well to a stretched exponential (exp[−*an* ^*b*^] with *a* = 0.44 ± 0.12 and *b* = 0.62 ± 0.01).

The number of news entries *n* per day is a stochastic variable, and often there is a burst of activity noted (Fig. 1a,b). The amount of data cataloged is also observed to have increased since the late 2000’s. The number of news entries/reports on a single day *n* has a broad distribution due to the large inter-day fluctuations in the number of reports and bursty nature of the data. Figure 1c shows the complementary cumulative distribution function (CCDF) that a day has more than *n* news reports, *Q*(*n*). For EC, the distribution has a short lognormal body with a rather prominent power law tail (decay exponent close to 2.54 ± 0.03); for HR, the bulk of the distribution fits to a stretched exponential (exp[−*an* ^*b*^] with *a* = 0.44 ± 0.12 and *b* = 0.62 ± 0.01). The details of the database, data extraction and different attributes are described in Supplementary Information. In our study, we focus on a relatively recent time span (2001–2015) and extract the data for actor pairs and geographical location of events from the database.

### Static properties of the network

**Figure 2:**
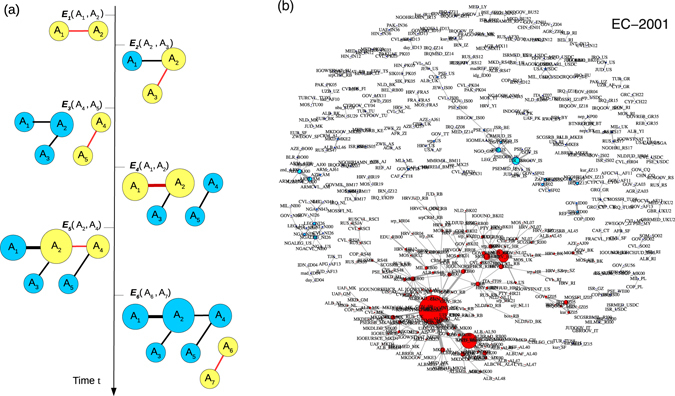
(**a**) The schematic visualization of events on a time-line and the subsequent evolution of the aggregated network of actors: Events appear sequentially. Event *E*_1_ involving actors *A*_1_, *A*_2_ is followed by event *E*_2_ involving *A*_2_, *A*_3_ and so on. The links depict the actors involved together in an event. New events (actors in yellow and links in red) add to actor mention (size of node) and actor co-mention (link weight; thickness of links). (**b**) The network of actors for EC for 2001. Each actor is a node and any two actors ever involved in an event are connected by a link. The size of the nodes are proportional to the total number of mentions in the period, and the relative thickness of the links are proportional to the total number of co-mentions of the actors (link weights). The largest connected component (giant component) is depicted in red.

We construct the aggregate network over a given period of time, for the entire 15 year period (2001–2015) as well as for each of the 15 individual years, for the two datasets. Each event is visualized as a link between the actors involved, which are the nodes, thus creating a network of actors connected by events. The details of the construction is given in the Methods section. A schematic representation of the network construction is given in Fig. 2a and a typical network is shown in Fig. 2b for EC data aggregated over the year 2001.

In our study, the temporal granularity of the data is one day. We extracted (i) the number of mentions *m* of each individual actor and (ii) the number of co-mentions *w* of an unique pair of actors (actor pair mentions), as well as the number of unique actors *k*, one actor is involved with. In terms of the network, *m* measures node strength, *w* the link weight and *k* the degree of a node. While *m* and *k* measure the importance, activity or visibility of a single actor, *w* measures the involvement of an actor pair in inciting an event. These quantities are measured for each year for the 15 year period (2001–2015) as well as through the whole period (aggregate over 15 years).

**Figure 3:**
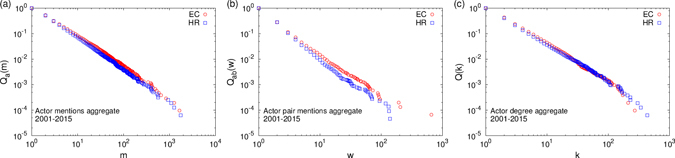
Structural properties of the aggregated network in the period 2001–2015: (**a**) Plot of the cumulative probability (CCDF) *Q*_*a*_(*m*) that an actor is mentioned at least *m* times. Except for an exponential decay at the very end of the tail, most of the distribution has a the power law decay ${Q}_{a}(m)\sim {m}^{-{\nu }_{1}}$ with exponents *ν*_1_ as 1.23 ± 0.01 for EC and 1.28 ± 0.01 for HR. (**b**) Plot of the cumulative probability *Q*_*ab*_(*w*) that an actor pair is mentioned at least *w* times. The tail of the distributions fit well to power laws ${Q}_{ab}(w)\sim {w}^{-{\nu }_{2}}$ with exponents as 1.58 ± 0.01 for EC and 1.74 ± 0.03 for HR. (**c**) Plot of the cumulative probability *Q*(*k*) that an actor is connected to *k* others or more. The tail of the distributions fit well to power laws $Q(k)\sim {k}^{-{\nu }_{3}}$ with exponents *ν*_3_ as 1.52 ± 0.01 for EC and 1.48 ± 0.01 for HR.

It is often perceived that certain actors frequently engage in reported events than others and the same is true for pairs of actors engaging in ethnic conflicts or human rights violations. We computed the complementary cumulative probability distribution (CCDF) for different quantities for the aggregated network in 2001–2015 (refer to Fig. 3a–c). For the actor mentions, the probability that an actor is mentioned *m* times or more fits to a power law for the largest values, ${Q}_{a}(m)\sim {m}^{-{\nu }_{1}}$, with decay exponents 1.23 ± 0.01 for EC and 1.28 ± 0.01 for HR. The exponents are computed, along with the values of the standard errors (uncertainties) and significances, using Maximum Likelihood Estimates^19^. The probability that an actor pair is mentioned *w* times or more fits well to power laws ${Q}_{ab}(w)\sim {w}^{-{\nu }_{2}}$ with exponents *ν*_2_ as 1.58 ± 0.01 for EC and 1.74 ± 0.03 for HR data. The *actor mentions* and *actor pair mentions* are respectively the *node weights* and *edge weights* in network terminology. The probability that an actor was involved with *k* or more actors during the time span (degree of the actor) fit well to power laws $Q(k)\sim {k}^{-{\nu }_{3}}$ with exponents *ν*_3_ as 1.52 ± 0.01 for EC and 1.48 ± 0.01 for HR data. For the individual years, due to less aggregation, the counts are less and data naturally seems noisy. However, the data still seem to exhibit the power law tail in the probability distributions (see Supplementary Fig. S1). The fitting exponents for the individual years are given in Supplementary Table S1. The correlation between actor degree and mentions, as well as the relationship between the power law exponents *ν*_1_ and *ν*_3_ are shown in Supplementary Fig. S2.

The above results quantitatively characterize the heterogeneity in the activity of actors, while most actors are relatively less active: the power law distributions for actor mentions *Q*_*a*_ indicate that there are a significant few who constantly engage in ethnic conflicts and human rights violations issues. The power law distributions in actor pair mentions *Q*_*ab*_ indicate similar characteristic for pairs of actors. The broad degree distributions *Q*(*k*) are indicative of the fact that the number of actors engaging with very large number of actors are also significant.

### Clusters

**Figure 4:**
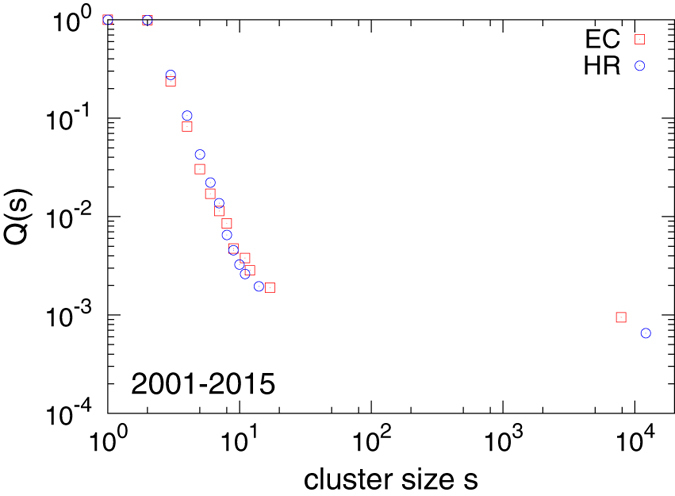
Plot of the cumulative probability (CCDF) *Q*(*s*) that there is a cluster of size larger than *s*, for data aggregated over the period 2001–2015. The size of the largest cluster is very large compared to the rest.

The aggregated network is found to be composed of several disconnected components or ‘clusters’. Physically this means that the actors constituting one cluster have never been involved with any actor from a different cluster. For both sets, the largest connected component is 10^2^–10^3^ times the smaller clusters, which are large in number (see Supplementary Table S2). In fact, the largest cluster *s*_1_ grows superlinearly with the size of the network *N*, as we found (from the data in Supplementary Table S2), ${s}_{1}\sim {N}^{\delta }$, with *δ* = 1.17 ± 0.01 for EC and 1.19 ± 0.02 for HR (Supplementary Fig. S3). This tells us that the fraction of nodes in the largest cluster grows with the total size of the network quite fast so that eventually the fraction of nodes outside the largest cluster will be negligible. We computed the CCDF *Q*(*s*) of cluster size *s* and find that it roughly has a power law decay for components except the largest one, with a decay exponent roughly close to 3 (see Fig. 4). The CCDF for individual years has been shown in Supplementary Fig. S4. The largest clusters in both sets have a slowly decaying clustering coefficient 〈*C*(*k*)〉 with degree *k* (see Supplementary Fig. S5).

### Network growth properties

In order to explore the dynamics of the process that leads to the broad distribution of the mentions and degrees, we investigate the dynamics of growth for these quantities over the span of 15 years (2001–2015). For good statistics, we ranked the actors according to the number of mentions and degrees, and analyzed the data for the top 10 actors.

We first measured the growth of the degrees and mentions for the top 10 actors of EC and HR data, with respect to the time *t* ^*^ when they were first mentioned. The long time behavior (asymptotic) indicates a growth law of (*t* − t*)^*β*^ with $\beta \simeq 3$.

**Figure 5:**
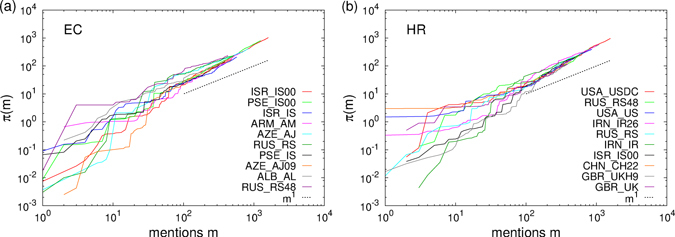
Cumulative growth rates *π*(*m*) for mentions *m* for (**a**) EC and (**b**) HR datasets. The curves asymptotically fit to $\pi (m)\sim {m}^{\alpha }$ with *α* > 1. The precise fitting exponents are given in Supplementary Table S4.

To extract the rate of growth for a quantity *x*, we computed ${\rm{\Pi }}(x)=\frac{{\rm{\Delta }}x}{{\rm{\Delta }}t}$. Π(*x*) in real data turns out to be very noisy, so we computed the cumulative integral $\pi (x)={\int }_{0}^{x}\,{\rm{\Pi }}(x^{\prime} )dx^{\prime}$ which turns out to be less noisy. We observe that data for degree *π*(*k*) has a shorter span and is noisier than that for mentions *π*(*m*). The plots suggest that both *π*(*m*) and *π*(*k*) are superlinear functions of their respective arguments, i.e., $\pi (x)\sim {x}^{\alpha }$ with *α* ~ 1.2–1.4 (see Fig. 5 for mentions and Supplementary Fig. S6 for degree). Thus the asymptotic growth rate ${\rm{\Pi }}(x)\sim {x}^{\alpha -1}$ is still weakly dependent on the respective arguments. We thus identify that the growth rate of the system (network) is not independent of the size of the node (degree or mentions). The growth exponents are computed using Maximum Likelihood Estimates^19^ and tabulated in Supplementary Table S4. A superlinear growth rate implies that the network will, in the long run, consist of a very large ‘condensate’ with a small fraction of nodes in isolated smaller clusters, which is consistent with our analysis of the cluster size distribution.

### Tolerance to attack and failure

**Figure 6:**
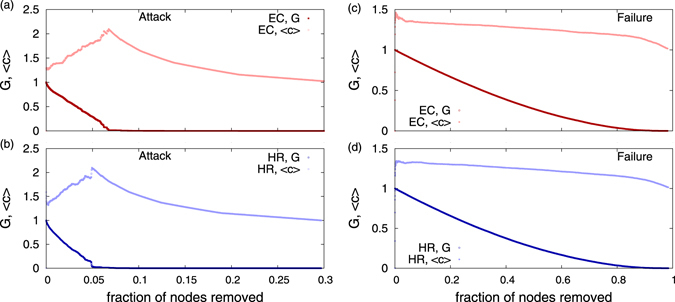
The structure of the network under attack: Nodes are removed in the sequence of their degrees starting from the highest degree. The plot shows the behavior of the giant component *G* (fraction of nodes in the largest connected component) and the average number of nodes in the clusters other than the giant component 〈*c*〉, with increasing fraction of removed nodes, for (**a**) EC and (**b**) HR. The structure of the network under random failure: Nodes are removed randomly. Results are shown for (**c**) EC and (**d**) HR networks. The networks are destroyed very quickly by targeted node removal (attack), compared to random node removal (failure). The results are for networks aggregated over 2001–2015.

We also study how the network breaks down under attack, in order to investigate the possibility of preventing unruly events to spread^20^. The largest connected component of the network is subjected to targeted attack by removal of the most connected nodes. We start by removing the node with the highest degree, followed by the next highest and so on. This results in rapid fragmentation or destruction of the network. We compute the fraction of nodes *G* present in the largest cluster, which is observed to decrease very quickly (Fig. 6a). In fact, the network of actors can be destroyed by targeted attack just by removing much less than 10% of the nodes compared to when randomly selected nodes are removed (random failure) one after another (Fig. 6b). This exercise indicates that targeted intervention may help stop spreading of ethnic conflicts and human rights violations.

### Measuring causality

Considering that we have time series data for the number of mentions of ethnic conflicts and human rights violations from news articles it would be interesting to study if there is a clear cause and effect relation between these events with time. To determine the causal effect purely from the observations of the past data, we apply Granger Causality^21^ which estimates the causal relationship by observing the changes in the distribution of the variables over time.

Let us consider two random variables depicting counts of EC and HR. To say that EC causes HR, Granger causality computes a regression of variable HR on the past values of itself and the past values of EC and then tests the significance of coefficient estimates associated with EC. We consider a bivariate linear autoregressive model on EC and HR, and assume the L.H.S. to be dependent on the history of EC and HR,

$$H{R}_{t}={a}_{0}+{a}_{1}E{C}_{t-1}+\cdots +{a}_{h}E{C}_{t-h}+{b}_{1}H{R}_{t-1}+\cdots +{b}_{h}H{R}_{t-h}+{E}_{t}$$

where *h* is the maximum number of lagged observations (for both EC and HR). The coefficients *a*_*i*_, *b*_*i*_ are the contributions of each lagged observation to the predicted value of *EC*_*t*−*i*_ and *HR*_*t*−*i*_ respectively and *E*_*t*_ is the prediction error.

We set up a null hypothesis and to test the significance of the coefficients, compute the *p* value. If the *p*-value is less than 0.05, one can reject the null hypothesis. We tested the counts for year wise as well as month wise mentions. Comparing the *p* values for both cases, we found that: (a) one cannot conclude that HR causes EC, but (b) EC causes HR. Hence, we can confirm that ethnic conflicts cause human rights violations, while human rights violations are not responsible for ethnic conflicts. The details of the analysis are provided in the Supplementary Information.

## Discussions

News reports serve as a reasonable proxy for the importance and intensity of events. The intensity is reflected by the number of reports and lingering span of time through which the reports follow. Our study focuses on events that pointed at ethnic conflicts and human rights violations. The GDELT data is unique in the sense that it records events and actors involved in them. The US government had recently funded in a large-scale project, the Integrated Conflict Early Warning System (ICEWS)^22^, which makes use of quantitative data and statistical methods in order to forecast events of political instability, which include international and domestic crises, ethnic and religious violence, rebellion and insurgency. We have chosen the GDELT data instead of the ICEWS data to get a more global perspective^23, 24^. The frequencies of mentions of actors and their co-mentions with others can be treated well as proxies for their importance and influence, as well as involvement with others. The aggregate data enables us to construct a network of actors, and even finding disconnected groups. In fact, our study reveals that most events are disconnected in very small clusters while very large clusters of frequently engaging actors exist. One can study the geographical localization of events and clusters to procure detailed information regarding the context, intensity and growth pattern of events^25^. Identifying important groups of actors, in terms of their intensities of activities, is important for possible intervention that may prevent the spread of such events. The probability distributions of actor mentions, co-actor mentions and the degree of an actor have power law tails for the largest values, indicating a strong self-organizing principle behind the events. The growth properties of individual actor nodes indicate that in the long run, very small fraction of disconnected clusters are left, while most of the actors belong to a giant connected component. The data on ethnic conflicts and human rights violations are found to be strikingly similar in terms of static and dynamic properties of the network, and even in terms of network stability against failure as well as targeted attack. This may point at a very high degree of correlation between events. In fact, using a causality analysis, we could quantitatively conclude that ethnic conflicts lead to human rights violations, while the reverse may not be true. These networks of ethnic conflicts and human rights violations reflect the negative aspects of human behavior and cooperation, and are certainly different from other social networks (friendship, collaboration, etc.) in the sense that there are not many triangles or closed communities, as observed in the distribution of the average clustering coefficient with degree of the actors. This detailed scientific study of the network structure, dynamics, function and resilience may help policy makers, specifically in cases where there is a need for preventing the spread of ethnic tensions and conflicts. There are possibilities of similar analyses using data from online social media, e.g., *Twitter*, etc.

We have deliberately avoided mentioning the real names and other details because the main aim of the paper was to study the network properties and statistical regularities. Since we have just considered the reports as a proxy for the events and not studied the reports in details to extract further information, it would not be proper to draw any conclusions about the actors, the network relations or the detailed explanations/causes behind the conflicts or the violations. Certainly, it would be interesting to address such sociological explanations, implications and policies in the future. Specific studies on the geographical implications of actor networks, the geographical and socio-cultural influence of their robustness under possible intervention will be important issues to study.

## Methods

### Data acquisition and filtering

GDELT Event Database^18^ contains the database of news articles from around the world in several languages, hosted through *Google Cloud*. Using *Google BigQuery* ^26^, it is possible to extract data for each event, having an unique time stamp, and providing the data about news about ethnic conflicts (EC) and human rights violations (HR) happening around the world spanning over a large time scale. The data contains information about a pair of *actors* involved, the location information of the event, as well as latitude, longitude data of actors and the event. We procured 45,942 events for EC and 48,295 for HR for a 15 year period, 2001–2015. We filtered out those data for which both actors were mentioned, along with their respective location information. The GDELT data has a huge fraction of missing entries. We have filtered out and excluded those data rows, which have at least one entry missing corresponding to the attributes we were interested in. Hence, after cleaning, we analyzed 28,055 events of EC and 36,470 of HR. Details of the CAMEO codes used for filtering the data (along with examples from the CAMEO codebook)^27^, data attributes and cleaning are provided in Supplementary Information.

### Network construction

Given a period of time *T*, we construct the network of ‘connected’ actors in the following way: any two actors *A*_1_ and *A*_2_ mentioned together in an event *E*_1_ reported at time *t* ∈ [*t*_0_ : *t*_0_ + *T*] are ‘connected’ by a link of unit weight,where *t*_0_ is the beginning of an interval of time span *T*. If another event *E*_2_ within the same time window involves actors *A*_2_ and *A*_3_, then *A*_3_ is connected to *A*_2_ with a link of unit weight. Thus *A*_1_ and *A*_3_ are both connected to *A*_2_ (see Fig. 2a). Aggregating all such events over the time window *T*, connected components emerge, with link weights increasing if the same pair of actors linking them appear in multiple events (actor pair mentions). These connected components form a complex network of nodes (actors) and links (actor pair mentions). An actor may be co-mentioned with several other actors and thus have a larger ‘degree’, measured by the number of distinct co-actors it has. In principle, the network aggregated over a time period can have several disconnected components or ‘clusters’. Figure 2b shows the aggregated network for one year for actors mentioned in the EC dataset.

## Electronic supplementary material

Supplementary Information
