# Supplementary material for: A complex network analysis of ethnic conflicts and human rights violations
Source: Sci Rep. 2017 Aug 15;7:8283. doi: 10.1038/s41598-017-09101-8 (PMC5557885; doi:10.1038/s41598-017-09101-8)
Supplement: Supplementary file 1 — Supplementary Information [file 41598_2017_9101_MOESM1_ESM.pdf]

Supplementary Information for "A complex network analysis of ethnic conflicts and human rights violations"

Kiran Sharma,[1] Gunjan Sehgal,[2] Bindu Gupta,[2] Geetika Sharma,[2]
Arnab Chatterjee,[2] Anirban Chakraborti,[1] and Gautam Shroff[2]

[1] *School of Computational and Integrative Sciences, Jawaharlal Nehru University, New Delhi-110067, India*
[2] *TCS Research, New Delhi, India*

Data Description

The data source for this quantitative analysis on the global structure of ethnic violence is GDELT, the Global Database of Events, Language, and Tone [1]. It is described [2] as "an initiative to construct a catalog of human societal-scale behavior and beliefs across all countries of the world, connecting every person, organization, location, count, theme, news source, and event across the planet into a single massive network that captures what's happening around the world, what its context is and who's involved, and how the world is feeling about it, every single day".

GDELT contains data since 1979 [3]. However, the data from the year 2000 onwards is more comprehensive, reflecting the increase in the number of news media and the frequency of event recording. The entire GDELT dataset is available as a public dataset in Google Big Query [4]. GDELT event records are stored in an expanded version of the dyadic CAMEO format, capturing two actors and the action performed by *Actor1* upon *Actor2*. A wide array of variables break out the raw CAMEO actor codes into their respective fields to make it easier to interact with the data, the *Action* codes are broken down into a hierarchical structure, a score describing the intensity of conflict or cooperation is provided, an average *tone* score is provided for all coverage of the event, several indicators of "importance" based on media attention are provided, and an unique array of geo-referencing fields offer estimated landmark-centroid-level geographic positioning of both actors and the location of the action.

However, for the purpose of this quantitative analysis we have extracted the data for the CAMEO codes 203 (engage in ethnic cleansing) for Ethnic Conflicts (EC) and 092 (investigate human rights abuses), 1122 (accuse of human rights abuses) for Human Rights Violations (HR) from the years 2001 to 2015.

As mentioned in the CAMEO codebook [5], for the corresponding entries, we reproduce below the cited examples:

- `Cameo code 203` **for EC:** Serb forces were engaged in ethnic cleansing in Kosovo against the majority Albanian population of the province, according to the US government.
- `Cameo code 092` **for HR:** Israel's high court opened a landmark hearing Wednesday into the legality of secret interrogation techniques used against Palestinian detainees.
- `Cameo code 1122` **for HR:** Human rights watchdog Amnesty International accused the United States of violating human rights, ignoring international law and sending a "permissive signal to abusive governments".

Each data entry with a *GLobalEventID* had the following attributes [5]:

- `Actor1Code, Actor2Code`: The complete raw CAMEO code for *Actor1* and *Actor2* (includes geographic, class, ethnic, religious, and type classes). It may be blank if the system was unable to identify any actor.
- `Actor1Name, Actor2Name`: The actual name of the *Actor1* and *Actor2*. In the case of a political leader or organization, this will be the leader's formal name (e.g., GEORGE W BUSH, UNITED NATIONS), for a geographic match it will be either the country or capital/major city name (e.g., UNITED STATES / PARIS), and for ethnic, religious, and type matches it will reflect the root match class (e.g., KURD, CATHOLIC, POLICE OFFICER, etc). It may be blank if the system was unable to identify an actor.
- `Actor1Geo_ADM1Code, Actor2Geo_ADM2Code`: This is the 2-character FIPS10-4 country code followed by the 2-character FIPS10-4 administrative division 1 (ADM1) code for the administrative division housing the landmark.
- `ActionGeo_FullName`: Location of Event. This is the full human-readable name of the matched location.
- `ActionGeo_CountryCode`: Location of Event. This is the 2-character FIPS10-4 country code for the location.
- `ActionGeo_ADM1Code`: Location of Event. This is the 2-character FIPS10-4 country code followed by the 2-character FIPS10-4 administrative division 1 (ADM1) code for the administrative division housing the landmark.
- `ActionGeo_Lat, ActionGeo_Long`: This is the centroid latitude and longitude of the landmark for mapping.

- `SQLDATE`: Date the event took place in *YYYYMMDD* format.

There may be actors with similar `ActorCode` at different locations. So, for uniquely identifying each actor we have concatenated `Actor1Code` with `Actor1Geo_ADM1Code` and `Actor2Code` with `Actor2Geo_ADM2Code`. The data acquired from queries had to further cleaned for missing entries. Each event entry mentions a pair of unique actors, but we also found rare instances ($< 0.5\%$) where only one actor has been identified. Any row of data with missing actor names, actor codes or location data were removed to created the working data set. The number of events were $45,942$ for EC and $48,295$ for HR and this was reduced to $28,055$ for EC and $36,470$ for HR after filtering. On actual inspection of the data, we found that a very small fraction of entries do not actually report an actual case of ethnic conflict or human rights violations, yet contain the relevant keywords that dicusss the issues in a positive tone (e.g. absence of ethnic violence or human right violations etc.).

The structure of the network

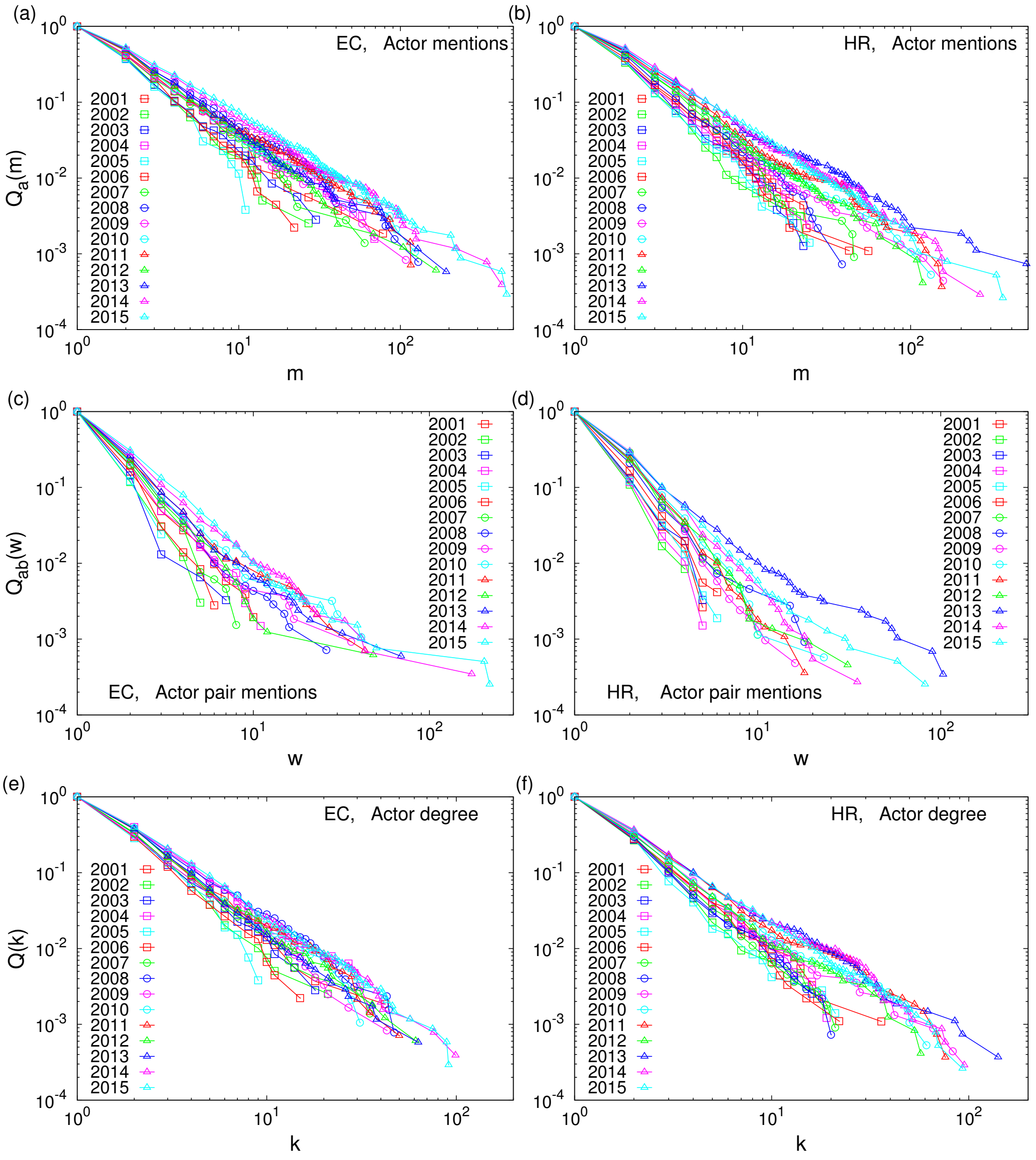

FIG. S1: Plots of the cumulative probability (CCDF) $Q_a(m)$ an actor is mentioned m times or more, for (a) EC and (b) HR; $Q_{ab}(w)$ that an actor pair is mentioned w times or more for (c) EC and (d) HR, and $Q(k)$ that an actor is co-mentioned with k actors or more for (e) EC and (f) HR, for each year in the period 2001-2015. The actual fits are given in Table S1.

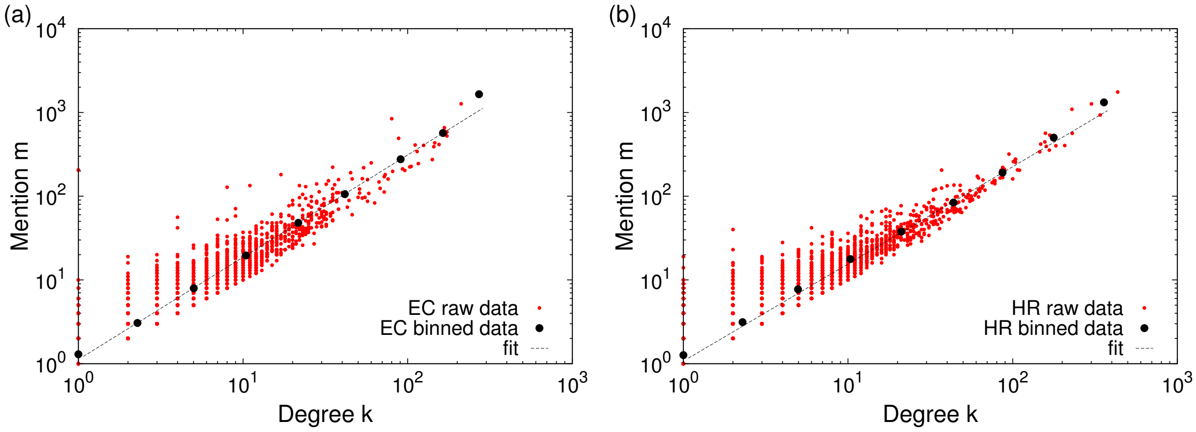

FIG. S2: Scatter plots showing the degree (k) versus mention (m) (red) for each actor, in the networks for (a) EC and (b) HR. The raw data (red) are then log-binned and plotted as black filled circles. The dashed lines are the best fits obtained using ordinary least squares for the log-binned data (black filled circles): $m \propto k^{\gamma}$ with γ measured to be 1.22 ± 0.01 for EC and 1.16 ± 0.02 for HR. The slope of each best fit line implies strong correlation between the degree (k) and mention (m) for actors, as also reflected by the ratios of the power-law exponents of the probability distributions of the respective variables in Fig. 3: $\nu_3/\nu_1 \approx 1.23$ for EC and ≈ 1.16 for HR, compares well to corresponding values of γ.

TABLE S1: Computed exponents power law distribution fits for (i) actor mentions, (ii) actor pair mentions and (iii) degree for the data – Ethnic Conflicts (EC) and Human Rights Violations (HR)for different years (2001-2015) as well as for the 15 year aggregate.

Year	Actor pair mention		Actor mention		Actor degree	
	EC	HR	EC	HR	EC	HR
2001	2.61 ± 0.07	3.47 ± 0.35	1.56 ± 0.05	1.79 ± 0.04	2.13 ± 0.05	2.01 ± 0.06
2002	3.47 ± 0.23	3.55 ± 0.22	1.90 ± 0.06	2.06 ± 0.07	2.44 ± 0.07	2.16 ± 0.09
2003	3.04 ± 0.37	3.32 ± 0.35	1.71 ± 0.03	2.03 ± 0.07	2.01 ± 0.05	2.15 ± 0.04
2004	2.53 ± 0.11	3.81 ± 0.39	1.42 ± 0.02	1.90 ± 0.04	1.66 ± 0.04	2.06 ± 0.03
2005	3.35 ± 0.24	3.48 ± 0.18	2.15 ± 0.11	2.08 ± 0.05	2.29 ± 0.15	2.16 ± 0.10
2006	3.25 ± 0.20	3.14 ± 0.17	2.15 ± 0.07	1.99 ± 0.08	2.24 ± 0.09	2.30 ± 0.07
2007	3.04 ± 0.18	3.04 ± 0.14	1.64 ± 0.04	1.86 ± 0.07	1.85 ± 0.05	2.25 ± 0.05
2008	2.27 ± 0.07	2.28 ± 0.13	1.43 ± 0.03	1.74 ± 0.04	1.69 ± 0.03	2.11 ± 0.05
2009	2.07 ± 0.17	2.97 ± 0.09	1.50 ± 0.02	1.54 ± 0.03	1.93 ± 0.04	1.69 ± 0.05
2010	1.47 ± 0.10	2.61 ± 0.15	1.30 ± 0.03	1.49 ± 0.02	1.71 ± 0.03	1.62 ± 0.04
2011	1.67 ± 0.07	2.81 ± 0.07	1.32 ± 0.01	1.38 ± 0.03	1.62 ± 0.04	1.56 ± 0.03
2012	2.86 ± 0.13	2.60 ± 0.13	1.54 ± 0.02	1.52 ± 0.02	1.80 ± 0.03	1.72 ± 0.04
2013	1.92 ± 0.07	1.41 ± 0.05	1.40 ± 0.02	1.21 ± 0.02	1.86 ± 0.02	1.57 ± 0.02
2014	1.83 ± 0.04	2.48 ± 0.05	1.29 ± 0.01	1.37 ± 0.01	1.69 ± 0.02	1.60 ± 0.03
2015	1.82 ± 0.06	2.06 ± 0.07	1.31 ± 0.01	1.45 ± 0.01	1.68 ± 0.02	1.64 ± 0.01
2001-2015	1.58 ± 0.01	1.74 ± 0.03	1.23 ± 0.01	1.28 ± 0.01	1.52 ± 0.01	1.48 ± 0.01

Clusters

TABLE S2: Table showing the number of actors and the actors in the largest connected cluster for the different data sets: Ethnic Conflicts (EC) and Human Rights Violations (HR) for different years (2001-2015) as well as for the 15 year aggregate.

Year	EC		HR	
	Total nodes	Biggest cluster size	Total nodes	Biggest cluster size
2001	540	193	917	333
2002	396	104	636	72
2003	355	117	788	184
2004	633	329	823	285
2005	263	48	714	110
2006	451	56	907	183
2007	721	288	1,102	309
2008	1,279	696	1,375	451
2009	1,204	544	2,267	1,011
2010	951	474	1,887	954
2011	1,412	746	2,719	1,591
2012	1,644	899	2,423	1,142
2013	1,728	892	2,707	1,576
2014	2,560	1,620	3,447	2,152
2015	3,432	2,229	3,816	2,322
2001-2015	10,394	7,875	15,899	12106

The size of the largest cluster is computed as the maximum number of nodes s_1 in the giant component or largest subgraph and shown in Supplementary Table. S2 and the variation of s_1 with the size of the entire network is shown in Fig. S3, and the asymptotic fit is found to be $s_1 \sim N^{\delta}$. The degree distribution of the largest cluster / giant component has a power law tail. The power law exponents for the asymptotic fits are given in Supplementary Table. S3.

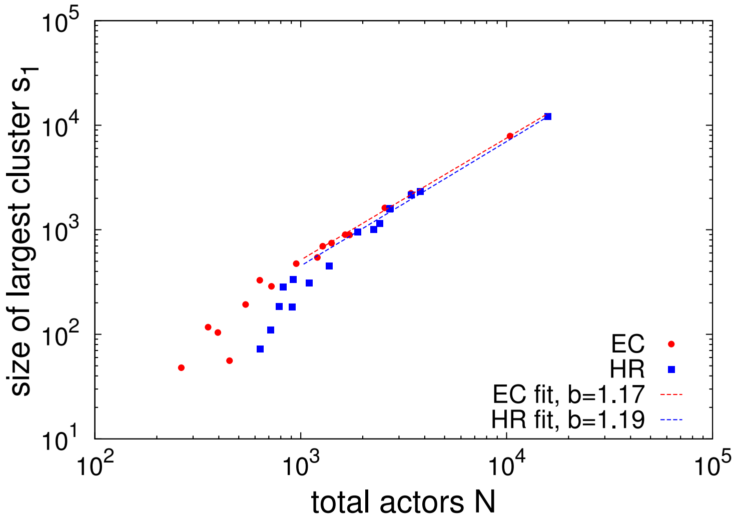

FIG. S3: Variation of the size of the largest cluster s_1 with network size N, for different years (2001-2015) and well as 15 year aggregate for EC and HR. The power law fits to $s_1 \sim N^{\delta}$ are $\delta = 1.17 \pm 0.01$ for EC and 1.19 ± 0.02 for HR.

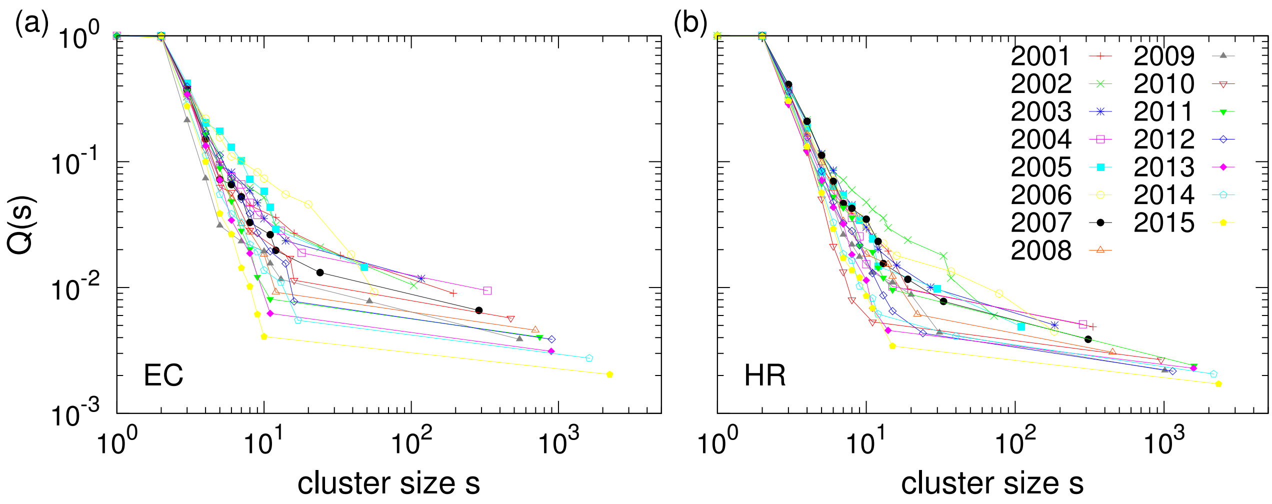

FIG. S4: Plots of the cumulative probability (CCDF) $Q(s)$ that there is a cluster of size larger than s, for different years (2001-2015) for (a) EC and (b) HR.

The size of the biggest cluster is computed as the maximum number of nodes in the giant component or a subgraph as shown in Table. S3 .

TABLE S3: Computed exponents ν of power law distribution to asymptotic fits to the degree distribution $Q(k) \sim k^{-\nu}$ for the largest connected cluster (giant component) for the two categories of data – Ethnic Conflicts (EC) and Human Rights Violations (HR) for individual years and the 15 year aggregate (2001-2015).

Year	EC	HR
2001	1.99 ± 0.06	1.71 ± 0.07
2002	1.84 ± 0.08	1.65 ± 0.05
2003	1.84 ± 0.08	1.56 ± 0.05
2004	1.58 ± 0.05	1.83 ± 0.06
2005	1.75 ± 0.29	1.48 ± 0.09
2006	1.50 ± 0.05	1.97 ± 0.08
2007	1.71 ± 0.06	1.84 ± 0.04
2008	1.64 ± 0.04	1.81 ± 0.08
2009	1.88 ± 0.05	1.56 ± 0.04
2010	1.62 ± 0.04	1.49 ± 0.03
2011	1.50 ± 0.03	1.41 ± 0.04
2012	1.75 ± 0.03	1.60 ± 0.04
2013	1.83 ± 0.02	1.53 ± 0.02
2014	1.67 ± 0.03	1.57 ± 0.04
2015	1.69 ± 0.02	1.60 ± 0.01
2001-2015	1.51 ± 0.01	1.48 ± 0.01

Biggest cluster: clustering coefficient

To find the cohesion of ethnic conflict and human rights violation, we computed the clustering coefficient of both (5 years aggregated) networks, which shows the extents to which the nodes of a network are closely connected with one another. The clustering coefficient for a node i in an undirected graph is computed as: $C_i = \frac{2N_i}{k_i(k_i-1)}$, where k_i is the degree of the node i, and N_i is the number of links between the neighbors of i. We plot the average clustering coefficient of a node with degree k in Fig. S5, and find that higher degree nodes are less clustered compared to low degree nodes.

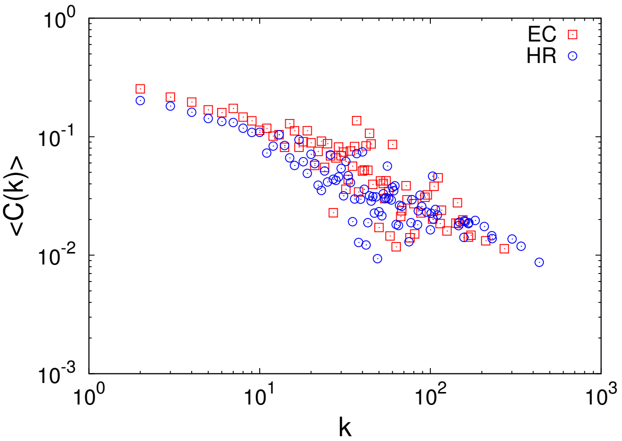

FIG. S5: Plot of average clustering coefficient $\langle C(k) \rangle$ with degree k of the biggest cluster for the two categories of data – EC and HR for the 15 years aggregate (2001-2015). The data for all sets exhibit a slow decay with degree.

Dynamics of network growth

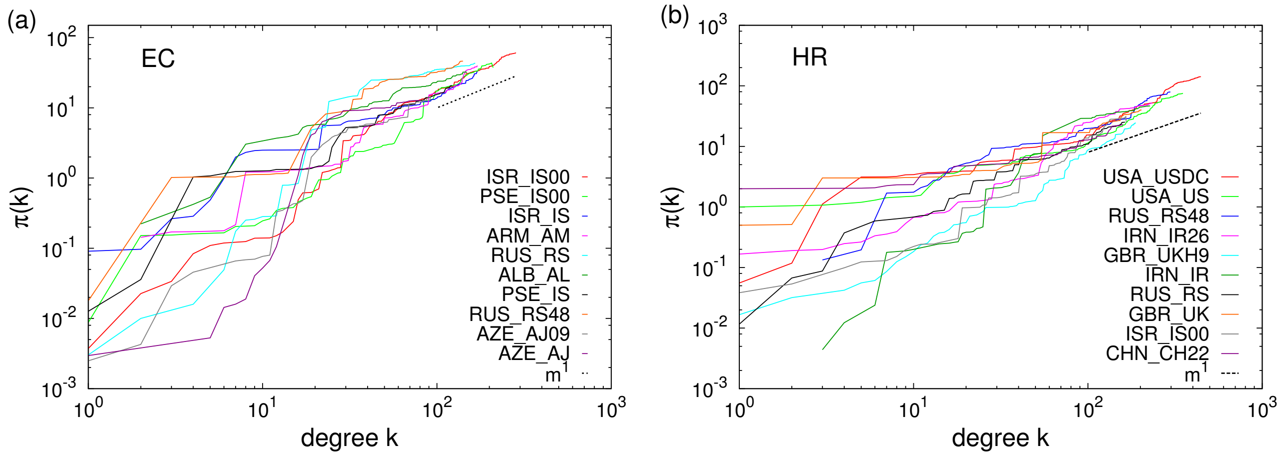

FIG. S6: Plots showing the cumulative growth rates $\pi(k)$ for degree k for (A) EC and (B) HR datasets. The curves fit to $\pi(k) \sim k^a$ with $a > 1$. The precise fitting exponents are given in Table. S4.

TABLE S4: Computed exponents of asymptotic power law fits for the cumulative growth rates $\pi(x) \sim x^a$ for the top ten highest counts for (i) actor mentions, (ii) degree, for the 2 categories of data – Ethnic Conflicts (EC) and Human Rights Violations (HR) for the 15 year aggregate.

EC			HR		
Actor	Degree	Mention	Actor	Degree	Mention
ALB_AL	0.86 ± 0.01	1.04 ± 0.01	CHN_CH22	0.71 ± 0.02	1.11 ± 0.01
ARM_AM	1.55± 0.02	1.51± 0.01	GBR_UK	1.11± 0.02	1.33± 0.01
AZE_AJ	0.50± 0.02	0.96± 0.01	GBR_UKH9	1.68± 0.02	1.79± 0.01
AZE_AJ09	0.96± 0.04	1.28± 0.01	IRN_IR	0.57± 0.01	1.10± 0.01
ISR_IS	0.89± 0.02	1.23± 0.01	IRN_IR26	1.58± 0.03	1.47± 0.01
ISR_IS00	1.26± 0.01	1.38± 0.01	ISR_IS00	1.86± 0.02	1.71± 0.01
PSE_IS	1.05± 0.02	1.31± 0.01	RUS_RS	1.27± 0.02	1.41± 0.01
PSE_IS00	1.82± 0.02	1.40± 0.01	RUS_RS48	1.04± 0.02	1.22± 0.01
RUS_RS	0.47± 0.01	0.92± 0.01	USA_US	1.33± 0.02	1.40± 0.01
RUS_RS48	0.53± 0.02	0.79± 0.01	USA_USDC	1.29± 0.01	1.35± 0.01

Measuring causality

Let us consider the two random variables depicting counts of EC (Ethnic Conflicts mentions) and HR (Human Rights Violation mentions). To say that EC causes HR, Granger causality [6] computes a regression of variable HR on the past values of itself and the past values of EC and then tests the significance of coefficient estimates associated with EC.

We consider a bivariate linear autoregressive model on EC and HR, and assume the L.H.S. to be dependent on the history of EC and HR,

$$HR_t = a_0 + a_1 EC_{t-1} + \ldots + a_h EC_{t-h} + b_1 HR_{t-1} + \ldots + b_h HR_{t-h} + E_t \quad (1)$$

where h is the maximum number of lagged observations (for both EC and HR). The coefficients a_i, b_i are the contributions of each lagged observation to the predicted value of EC_{t-i} and HR_{t-i} respectively while E_t is the prediction error.

If $b_1 = b_2 = \ldots = b_h = 0$, we call it a null hypothesis HR_0 which implies that EC does not cause HR. In other words the coefficients of EC are not significant enough to cause HR. But if the null hypothesis gets rejected we say that the coefficients of EC are significant enough to cause HR.

For testing this significance of the coefficients, we compute the p-value. If the p-value is less that 0.05 one can reject the null hypothesis, and hence conclude that EC causes HR (HR $\sim$ EC).

Applying the above process on the EC and HR *year wise* mentions, we found that:

- On testing $EC \sim HR$ for $h = 4$, we find $p \simeq 0.721$, and the null hypothesis cannot be rejected. Hence, one cannot conclude that HR causes EC.
- On testing $HR \sim EC$ for $h = 4$, we find $p \simeq 0.029$, and the null hypothesis can be rejected. Hence, we can say that EC causes HR.

Applying the above process on the EC and HR *month wise* mentions, we found that:

- On testing $EC \sim HR$ for $h = 5$, we find $p \simeq 0.193$ and thus the null hypothesis cannot be rejected. Hence, we cannot say that HR causes EC.
- On testing $HR \sim EC$ for $h = 5$, we find $p \simeq 0.036$, and thus the null hypothesis can be rejected. Hence, we can say that EC causes HR.

Hence, we can definitely conclude from the above quantitative analysis that Ethnic conflicts cause Human Rights violations.

[1] The GDELT Project, retreived October, 2016. `www.gdeltproject.org/`.
[2] K. Leetaru and P. A. Schrodt. Gdelt: Global data on events, location, and tone, 1979–2012. In *ISA Ann. Convention*, volume 2. Citeseer, 2013.
[3] J Dana Stuster. Mapped: Every protest on the planet since 1979. *ForeignPolicy. com*, 2013.
[4] World's largest event dataset now publicly available in BigQuery. `https://cloudplatform.googleblog.com/2014/05/worlds-largest-event-dataset-now-publicly-available-in-google-bigquery.html`.
[5] GDELT - DATA FORMAT CODEBOOK V 1.03, as on 8/25/2013. `http://data.gdeltproject.org/documentation/GDELT-Data_Format_Codebook.pdf`.
[6] C. W. J. Granger. Investigating causal relations by econometric models and cross-spectral methods. *Econometrica*, 37:424–438, 1969.
